# Exploring Different Levels of Contact Frequency in Multiple Sclerosis Care

**DOI:** 10.1002/brb3.70634

**Published:** 2025-07-07

**Authors:** Sofia Persson, Ann‐Christine Andersson, Boel Andersson Gäre, Jonas Lind

**Affiliations:** ^1^ Jönköping Academy for Improvement of Health and Welfare, School of Health and Welfare Jönköping University Jönköping Sweden; ^2^ Futurum Academy for Health and Care Region Jönköping County Jönköping Sweden; ^3^ Department of Public Health and Health Care Region Jönköping County Jönköping Sweden; ^4^ The Child Health Care Service Region Jönköping County Jönköping Sweden; ^5^ Section of Neurology, Department of Internal Medicine County Hospital Ryhov Jönköping Sweden; ^6^ Division of Neurobiology, Department of Biomedical and Clinical Sciences Linköping University Linköping Sweden

**Keywords:** care contacts, contact frequencies, multiple sclerosis care

## Abstract

**Introduction:**

Previous studies have identified differences in healthcare contacts, needs, and cost of care among persons living with multiple sclerosis (MS). The need for a deeper understanding of factors influencing healthcare contacts has been highlighted. The aim of this study was to explore different levels of healthcare contact frequency among persons living with MS.

**Method:**

Both quantitative and qualitative data were collected, analyzed, and integrated in a parallel mixed‐methods approach with data integration through joint display. Data were retrieved from the hospital administrative system, the Swedish national MS registry, and a previously conducted interview study. The population was divided into four segments based on healthcare contact frequency, ranging from Segment 1, with the fewest visits, to Segment 4, representing those with the most frequent contacts. Analyses were conducted using descriptive statistics, statistical tests on differences between segments, multinomial logistic regression, deductive content analysis, and integration.

**Results:**

The mean and median distribution of individual study variables increased or decreased (depending on scale direction) between segments for most variables toward more symptoms, reduced function, and declining experiences of health from the lowest to the highest contact frequency. The probability of belonging to a certain segment was influenced by the explanatory variables age, gender, overall health, and expanded disability status with the different variables playing different roles for each segment. Qualitative findings also suggested increased physical limitations with increased level of healthcare contacts. This was not necessarily due to MS and influence of comorbidities was sometimes expressed. Both requests of less and more healthcare contacts existed and content of healthcare contacts could have more personalized design overall, health care was perceived positively across all segments. Data integration with merged interpretation included eight themes, characteristics of the population, neurological assessment and gait function, symptoms and consequences, fatigue and cognition, perspectives on health, examinations, interactions with health care, and disease duration and future. The merged interpretation confirmed patterns of symptoms, reduced function, and declining experiences of health from the lowest to the highest contact frequency and expanded on individual variation within segments and influenced by comorbidities. Discord in data regarded relations with others, aspects of medication, and knowledge building and type of MS.

**Conclusion:**

The findings on distribution of variables and experiences across segments, were increase in symptoms, loss of function, and deterioration of health experience correlating with increased levels of contact frequency from Segments 1 to 4. The explanatory variables found were age, gender, overall health, and expanded disability. The merged interpretation, expand on individual differences on how symptoms were experienced and influenced by comorbidities. Discord found, regarding for example personal context and aspects of self‐care where areas that may be overlooked in healthcare contacts. The explanatory variables identified in this study are suggested to be further explored together with the knowledge of persons living with MS and professionals.

## Graphical abstract

Segments representing contact frequencies.

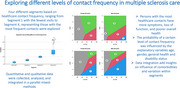

## 1 Introduction

Multiple sclerosis (MS) is a chronic autoimmune disease (Walton et al. 2020) with a high nationwide prevalence in Sweden, estimated at 189 cases per 100,000 individuals (Ahlgren et al. 2011). Symptoms are loss of neurological functions, pain, and fatigue (Browne et al. 2014; Nelson et al. 2019; Thompson et al. 2018; Ziemssen 2009), and the disease can influence quality of life (Chruzander et al. 2015; Isaksson et al. 2005). Over the past 25 years, life expectancy for individuals with MS has increased (Lunde et al. 2017; Marrie 2019), and the development of increasingly effective therapies has improved prospects for lives free from disability (Tanasescu et al. 2014). Chronic conditions such as MS differ from acute or temporary conditions in terms of the patients' evolving knowledge over time, emphasizing the importance of patients and health professionals sharing complementary knowledge (Holman and Lorig 2004). Differences exist between clinicians' and patients' views on what is important in MS care (Rothwell et al. 1997; Ziemssen 2009).

Patients with MS have been reported to have a high use of primary care and of neurology and rehabilitation departments, both in open care and as inpatients (Chruzander et al. 2015). The reason for contacting care is usually expected to be MS, but due to limitations in data (e.g., on comorbidities) it could also be related to other conditions or factors (Carlson and McGinley 2022; Marrie et al. 2015). Studies examining the experiences of individuals living with MS in various contexts and healthcare settings have highlighted the burden of illness, the progressive variation in care needs, and the necessity for care institutions to adapt their services over time (Borreani et al. 2014; Galushko et al. 2014; Hunter et al. 2021; Methley et al. 2017).

Previous studies have found considerable differences in care contacts and costs and that individual preferences and symptoms influence the timing, frequency, and preferred location for consultations with health care among those living with MS (Lind et al. 2022; Persson et al. 2023). Contributors to healthcare utilization in this population are generally understudied (Carlson and McGinley 2022). The need for deeper insight into factors influencing healthcare utilization and the diverse needs of persons living with MS to improve care has been emphasized (Bartolomeu Pires et al. 2024; Borreani et al. 2014; Carlson and McGinley 2022; Galushko et al. 2014; Hunter et al. 2021; Lind et al. 2022; Methley et al. 2017; Persson et al. 2023) and perspectives of lived experience can contribute to insights about the disease and about the medical care received (Given 2008).

Building on a previous study that identified four segments representing varying levels of healthcare contact frequencies ranging from Segment 1, with the fewest visits, to Segment 4, representing those with the most frequent contacts (Lind et al. 2022), the aim of this study was to explore these varying levels. Deeper insight into factors influencing healthcare contact may be used to improve MS care.

This population‐based study took place in Region Jönköping County in Sweden, where health care is primarily financed through general taxation, with small amounts of co‐payment for most types of health care. The Region has a long tradition of quality improvement, including coproduction work (Bodenheimer et al. 2007; Gäre and Neuhauser 2007; Persson et al. 2021; Staines et al. 2015).

The research questions were:

a) Are there differences in the variables including demographic data, disease specific data, and clinical data between the varying levels of contact frequency, and if so, what differences are found? (Quantitative)
  b) Which explanatory variables predict the level of contact frequency, and how are probabilities of having different levels of contact frequency influenced by these variables? (Quantitative)
What aspects of lived experiences are described by individuals with varying levels of contact frequency? (Qualitative)
What, if any, additional insights are gained through the integration of qualitative and quantitative data? (Quantitative and qualitative)

## 2 Materials and Methods

### 2.1 Design

A parallel mixed‐methods design was used, including separate collection and analysis of quantitative and qualitative data (Schoonenboom & Johnson 2017; Johnson 2007). This was followed by a merged interpretation giving equal value to the different data (Creswell and Plano Clark 2007), with a pragmatic underpinning (Baert 2004; Bryman 2006; Creswell and Plano Clark 2007).

### 2.2 Sampling

All individuals with diagnoses of MS (International Classification of Diseases 10 code G35) at the Section of Neurology at the Department of Internal Medicine at Ryhov Hospital between January 1, 2013 and December 31, 2021 were included in the initial quantitative sampling process (*n* = 431). Patients who only had one visit to the clinic during the time period (*n* = 20) as well as those who did not reside in the catchment area and had no registration in the MS‐registry were excluded (*n* = 6), as these groups likely received care elsewhere. This resulted in 405 patients for whom data were retrieved from the hospital administrative system (concerning contacts, age, disease duration, and gender). Of these patients, 274 were women and 131 were men, with a mean age of 48 years and a mean disease duration of 13 years.

Different levels of contact frequency were created by dividing the population (*n* = 405) into thirds, based on their average number of contacts. Contacts were defined as physical or digital consultations in primary care and hospital‐based care in the region and data was retrieved from the healthcare administrative system. The top third, consisting of patients with the most contacts, was divided into two equal groups, resulting in four segments. This process was informed by patterns identified in our previous study (Lind et al. 2022) and yielded the following segments: Segment 1 = 0 to < 8 mean contacts/year (*n* = 133); Segment 2 = 8 to < 16 mean contacts/year (*n* = 133); Segment 3 = 16 to < 23 mean contacts/year (*n* = 70); and Segment 4 = 23 to highest, mean contacts/ year (*n* = 69).

Quantitative data were additionally obtained from the Swedish national MS registry (Alping et al. 2019; Hillert and Stawiarz 2015). The registry includes patient characteristics, MS disease data, therapies, contacts, clinical scales, relapses, magnetic resonance imaging (MRI), and laboratory tests (Alping et al. 2019). Data are collected in an unselected MS population during routine clinical visits, with the exception of relapses and MRI, which are recorded at the time they occur. There are no reimbursements linked to data entry, and participation is voluntary for both patients and neurologists. The coverage rate in Region Jönköping County in 2019 was 77.3% based on estimated prevalence. The selection of variables from the MS registry in our study was made by clinicians and the research team beforehand, based on clinical experience and variables often used in MS clinical studies that included both professional and patient‐reported data. Variables retrieved from the registry were: type of MS (relapsing‐remitting [RR], secondary progressive [SP], and primary progressive [PP]); Expanded Disability Status Scale (EDSS); MS Impact Scale (MSIS 29); Fatigue Scale for Motor and Cognitive functions (FSMC); Symbol Digit Modalities Test (SDMT); EQ‐VAS (Overall health from EuroQoL‐5 Dimension Questionnaire); “MS‐kollen” (Swedish version of Guy's Neurological Disability Scale [GNDS]); 6‐minute walking test (6‐min WT); Number of relapses; and new T2‐enhancing lesions on MRI T2. The variables are further described in Appendix S1 and include information on 353 of the 405 individuals from the initial sampling (85%). The number of patients with reported results in the different variables ranged from 153 to 353 (Table 1). The variation in MS registry data is attributed to differences in reporting practices at the clinic.

**TABLE 1:** Number of patients with reported results for each variable in the MS registry, reported per segment and in total.

Variable	Segment 1		Segment 2		Segment 3		Segment 4		Total n
	*n*	%	*n*	%	*n*	%	*n*	%	
MS‐type	120	34	107	31	60	17	63	18	350
EDSS	117	35	105	31	59	17	58	17	339
MSIS	57	28	70	34	40	20	37	18	204
FSMC	41	27	51	33	32	21	29	19	153
SDMT	70	30	74	32	45	19	43	19	232
EQ‐VAS	70	29	80	33	44	18	47	20	241
“MS‐kollen”	45	28	54	34	32	20	29	18	160
6‐min WT	61	32	61	32	36	19	33	17	191
Relapses	121	34	109	31	60	17	63	18	353
MRI T2	104	34	97	31	55	18	52	17	308

*Note*: See variable explanations in the text.

The final model in of the multinomial logistic regression model included *n* = 182 (Table 2).

**TABLE 2:** Description of number of persons included in the multinomial logistic regression by segment and gender.

	Male	Female	Total
Segment 1	22	28	50
Segment 2	17	44	61
Segment 3	11	27	38
Segment 4	6	27	33
Total	56	126	182

Qualitative data were obtained from our previous interview study (Persson et al. 2023), which included 10 persons living with MS from the four segments (Table 3). Five men aged 43−67 and five women aged 30−71 participated. The interviews took place in the first quarter of 2021. A semi‐structured interview guide was developed based on experiences of exploring lived experiences in quality improvement including coproduction (Persson et al. 2023). One structured question on health assessment of the state of health in the last year was included, measured on a five‐point scale from *very good* to *very bad*. Two pilot interviews were conducted to develop skills and understanding of interactions, and some questions were rephrased in coproduction between the persons with patient experience, professionals, and researchers. Seven persons chose to be interviewed through a digital video tool, recorded as video files, and three chose face‐to‐face interviews at the hospital, which were audio‐recorded. The interviews lasted 47 min on average and 390 min in total (with a variation from 22 to 55 min), and were recorded and transcribed verbatim.

**TABLE 3:** Description of persons included in the interview study in the four segments.

	Segment 1	Segment 2	Segment 3	Segment 4
*N* interviews	3	2	2	3
Age	37–47	61, 67	44, 46	31–71
Male (M)/female (F)	2 M/1 F	2 M/0 F	1 M/1 F	0 M/3 F

### 2.3 Analysis

#### 2.3.1 Quantitative Analysis

Quantitative data were analyzed using SPSS. For patients with more than one data point in each variable during the study period, a mean value was calculated. Age was defined as the individual's age in 2017, which represents the midpoint of the data sampling period. Each variable was analyzed using descriptive statistics and was explored in relation to valid data, outliers and extreme values, and assumptions for the statistical tests (Harrison et al. 2021). Descriptive statistics for categorical data are presented in numbers and percentages, and scale data in mean, median, standard deviation (SD), range, and inter‐quartile range (IQR) per segment. Differences in each of the included variables across segments were analyzed using graphs and statistical tests: one‐way analysis of variance (ANOVA) for normally distributed data, the Kruskal–Wallis test when the normality assumption was not met, and chi‐square tests for categorical data. Effect sizes were calculated using eta squared (*η* ^2^) for ANOVA, Phi for chi‐square tests, and epsilon squared (*ε* ^2^) for Kruskal–Wallis (Fritz et al. 2012; Harrison et al. 2021) (Appendix S3).

Multinomial logistic regression was used to analyze how explanatory variables influence the levels of contact frequency. No multicollinearity was found with Variance Inflation Factor threshold at 10. The stepwise backward method was used to start from a full model, but also to isolate a subset of these based on their ability to explain segment belonging. The possible explanatory variables were reduced in steps by backward elimination, where the nonsignificant variables with the highest *p* value were excluded in each step until only significant variables remained (Allison 2012). Variables with less than 200 registrations and/or outliers and extreme values across all segments were excluded.

From the coefficients in the regression model, estimated probabilities were calculated per segment, with support from a statistician. Conditions were defined for the variables gender (male/female) and age (25 and 65 years, respectively) and not for the other two variables (EQ‐VAS and EDSS). The conditions defined were used as examples to visualize the regression model. Probabilities were controlled in SPSS using estimated response probabilities.

The significance level was set at *p* = 0.05 in all tests and the confidence level at 95% for all confidence intervals (CI).

#### 2.3.2 Qualitative Analysis

The interviews were analyzed with deductive content analysis (Elo and Kyngäs 2008; Kyngäs et al. 2020). The analysis framwork used included themes from a previous inductive analysis of the interviews (Appendix S2) (Persson et al. 2023). S.P. conducted the deductive analysis in collaboration with A.C.A., an experienced qualitative researcher. All interviews were reread and analyzed segment by segment to get a sense of the data in relation to the different segments. Data extracts were sorted in the framework by segments for comparison of content (Appendix S2). The analysis and results were discussed among all authors in a reflective process.

#### 2.3.3 Merged Interpretation

Qualitative and quantitative data were analyzed separately and then merged using integration through joint displays. Bringing data together by organizing related data in a figure, table, matrix, or graph aims at visualizing insights beyond the information gained from the separate quantitative and qualitative results (Fetters et al. 2013; Guetterman et al. 2015). Quantitative results and qualitative subthemes were clustered and analyzed together. For example, the variables “MS‐kollen” and MSIS addressing symptom assessment and effects of MS were analyzed with the subtheme of symptoms and consequences from the qualitative analysis. The clusters were merged under shared themes and visualized in a table including both graphs and text from the perspectives of confirmation, expansion, or discord (Fetters et al. 2013). Data (subthemes or variables) that could not be integrated were presented under the theme “Discord in data.”

#### 2.3.4 Ethical Considerations

The study was approved by the Swedish Ethical Review Authority (Dnr 2022‐04954‐0) based on the Swedish Act (2003:460) on ethical review of research involving humans. This means that the research was reviewed in accordance with the Declaration of Helsinki and relevant research guidelines.

For the quantitative data, the individual's social security number was required in order to link register data with the first sampling. Immediately after making the connection, the social security number was removed and replaced by a code. This means that the risk of identities being jeopardized is minimal. All data processing took place without a connection to social security numbers. In “Information to patients about the Swedish neuro registry,” it is communicated that registry data can be used for research after ethical approval.

Participation in interviews was voluntary, and choosing not to participate entailed no negative consequences. Reasons for not wanting to participate or for cancelling participation did not need to be stated. Written and oral information about the study was provided and opportunities were given to ask clarifying questions. Written informed consent was obtained. Findings were reported in a manner that limits the risk of identifying participants.

## 3 Results

### 3.1 Descriptive Statistics and Differences in Variables Across Segments

Descriptive statistics for categorical data (gender and type of MS) are presented by number and percentages (Table 4). Descriptive statistics for variables with scale data are presented with mean, median, SD, range, and IQR (Table 4).

**TABLE 4:** Distribution of the categorical data—gender and type of MS—in the four segments in numbers and percentages.

Segment	Men (*n* = 131)		Woman (*n* = 274)		RR (*n* = 232)		SP (*n* = 79)		PP (*n* = 39)	
	*n*	% within segment	*n*	% within segment	*n*	% within segment	*n*	% within segment	*n*	% within segment
Segment 1	56	42%	77	58%	88	73%	13	11%	19	16%
Segment 2	36	27%	97	73%	76	71%	24	22%	7	7%
Segment 3	21	30%	49	70%	33	55%	23	38%	4	7%
Segment 4	18	26%	51	74%	35	56%	19	30%	9	14%

Abbreviations: PP = primary progressive, RR = relapsing‐remitting, SP = secondary progressive.

There were more women than men across all segments, but in Segment 1 the distribution between men and women was more even. The RR type of MS was the most common across all segments, while the proportion of SP gradually increased along with contact frequency from Segments 1 to 4 (Table 4).

Variables measuring function, symptoms, and overall health (EDSS, FSCM, MSIS, MS‐kollen, SDMT, 6‐min WT, and EQ‐VAS) increased or decreased (depending on scale direction) in mean and median along with increased levels of contact frequency (Table 5). Significant differences (*p* < 0.05) between segments were found in EDSS, SDMT, FSMC, MSIS, “MS‐kollen,” EQ‐VAS, gender, and type of MS. No significant differences between segments were found for age and disease duration (Appendix S3).

**TABLE 5:** Descriptive statistics for variables with scale data across segments.

	Segment 1					Segment 2					Segment 3					Segment 4				
Variable	Mean	Median	SD	Range	IQR	Mean	Median	SD	Range	IQR	Mean	Median	SD	Range	IQR	Mean	Median	SD	Range	IQR
EDSS	2.3	1.6	2.3	9	(0.9–3.5)	2.5	1.9	2.3	9	(0.7–3.4)	3.5	2.7	2.3	8.2	(1.8–6.0)	3.9	3.4	2.4	9	(1.8–6.0)
MSIS phys	10.7	7.5	9.9	40	(2.8–19.1)	24.0	14.5	23.0	87	(6.8–34.6)	32.2	33.5	20.8	74.5	(16.8–47.9)	36.6	36.0	18.3	82	(22.0–49.0)
MSIS psych	17.7	13.6	14.9	69.5	(5.2–28.0)	32.9	30.1	23.4	97	(12.3–50.0)	35.9	35.2	19.4	90.8	(24.2–43.9)	45.2	47	19.7	65	(25.3–64.0)
FSMC cog	20.9	19.0	10.0	36	(11.8–27.5)	25.8	26.0	10.8	36	(17.0–34.0)	30.3	31.6	10.3	38.5	(24.5–36.8)	31.7	31.0	9.2	36	(25.5–38.0)
FSMC motor	21.3	20.5	9.9	33.0	(11.8–28.8)	25.2	24.0	10.7	39.0	(16.0–34.0)	31.4	33.1	9.7	37.3	(26.5–38)	33.8	34.0	10.1	36.0	(27.5–40.7)
SDMT	52.8	55.0	10.3	52.5	(46.5–59.6)	50.5	51.6	12.0	59.0	(42.4–58.3)	49.7	49.0	10.2	40.8	(42.6–58.5)	45.1	43.3	16.3	76.1	(33.0–57.0)
EQ‐VAS	76.5	77.5	15.3	70	77.5(70.9–88.3)	70.9	74.0	17.1	78.3	(59.3–84.7)	61.9	60.9	17.9	73.75	(52.4–74.5)	56.9	57.5	18.5	87	(43.6–72.2)
“MS‐kollen”	6.4	4.6	4.9	20	(3.0–9.0)	8.9	6.8	6.9	29.8	(4.2–12.8)	11.8	11.8	5.7	22	(8.0–16.6)	13.5	14.0	5.9	29	(10.3–17.0
6‐min WT	579.4	620	146.1	791.0	(509.6–681.7)	580.2	594.0	105.4	690	(524.0–637.0)	477.8	522.6	167.6	715	(404.1–593.3)	433.1	480	168.2	647	(304.8–574.6)
Relapses	0.55	0.0	0.88	4	(0.0–1.0)	0.6	0.0	0.98	4	(0.0–1.0)	0.7	0.0	1.1	5	(0.0–1.0)	0.4	0.0	0.8	3	(0.0–1.0)
MRI T2	1.4	0.0	2.3	11	(0.0–2.0)	2.2	0.0	5.0	24	(0.0–2.0)	1.3	0.0	2.9	18	(0.0–2.0)	1.9	0.0	3.6	20	(0.0–3.0)
Disease duration	13.29	11.0	10.1	51	(5.0–20.0)	11.8	8	10.7	49	(4.0–18.5)	12.53	9.5	9.6	46	(6.0–18.25)	13.4	10	10.7	41	(3.5–23.5)
Age	49	50	14.5	72	(40.5–62.0)	46	44	16.5	75	(33.0–60.0)	46	45	15	66	(34.3–57.0)	50	52	14.7	65	(38–61)

Abbreviations: “MS‐kollen” = Swedish version of Guy's Neurological Disability Scale; 6‐min WT = 6‐minute walking test; EDSS = Expanded Disability Status Scale; EQ‐VAS = Overall health from EuroQoL‐5 Dimension Questionnaire; FSMC = Fatigue Scale for Motor and Cognitive functions; MRI T2 = new T2‐enhancing lesions on MRI; MSIS = MS Impact Scale 29; Relapses = Number of relapses; SDMT = Symbol Digit Modalities Test;

There was no difference in disease duration, new T2 enhancing lesions, or relapses between segments.

Visualizations of variables (as boxplots and bars) can be found in Appendix S4.

### 3.2 The Variables' Influence and Probabilities

The stepwise backward multinomial logistic regression included nine variables (gender, EDSS, type of MS, MSIS phys, MSIS psych, SDMT, EQ‐VAS, disease duration, and age) on the response variable (segments). The final model included four significant explanatory variables across the segments: gender, EDSS, EQ‐VAS, and age (Table 6). The model was statistically significant, *χ* ^2^_12_ = 71.568, *p* = < 0.001, *n* = 182.

**TABLE 6:** Final model from stepwise backward multinomial logistic regression including coefficient, standard error, odds ratio, confidence interval for odds ratio, and *p* value.

Explanatory variable	Coefficient (*B*)	Standard deviation (SD)	Odds ratio (OR)	95% CI for OR	*p* value
**Segment 2 vs. Segment 1**					
**Intercept**	4.137	1.570			0.008
**EDSS**	0.508	0.206	1.662	(1.110, 2.489)	0.014
**Gender (M = 1)**	−1.003	0.447	0.367	(0.153, 0.881)	0.025
**Age**	−0.053	0.020	0.948	(0.912, 0.986)	0.007
**EQ‐VAS**	−0.028	0.016	0.973	(0.942, 1.004)	0.085
**Segment 3 vs. Segment 1**					
**Intercept**	4.891	1.732			0.005
**EDSS**	0.814	0.220	2.257	(1.466, 3.476)	< 0.001
**Gender (M = 1)**	−1.280	0.553	0.278	(0.094, 0.822)	0.021
**Age**	−0.075	0.024	0.928	(0.886, 0.971)	0.001
**EQ‐VAS**	−0.043	0.018	0.958	(0.925, 0.995)	0.018
**Segment 4 vs. Segment 1**					
**Intercept**	5.893	1.762			< 0.001
**EDSS**	0.731	0.226	2.077	(1.33, 3.236)	0.001
**Gender (M = 1)**	−2.013	0.646	0.134	(0.038, 0.474)	0.002
**Age in 2017**	−0.059	0.024	0.943	(0.899, 0.989)	0.015
**EQ‐VAS**	−0.066	0.019	0.936	(0.902, 0.972)	< 0.001

The probabilities of belonging to different segments and thus having different levels of contact frequency are visualized in Appendix S3 and Figure 1. Probability of belonging to a given segment for men and women depending on EQ‐VAS and EDSS scores at ages 25 and 65 are visualized in Appendix S3. Figure 1 visualizes the probabilities by showing which segment has the highest probability in a certain area, for men and women, at ages 25 and 65.

**FIGURE 1:**
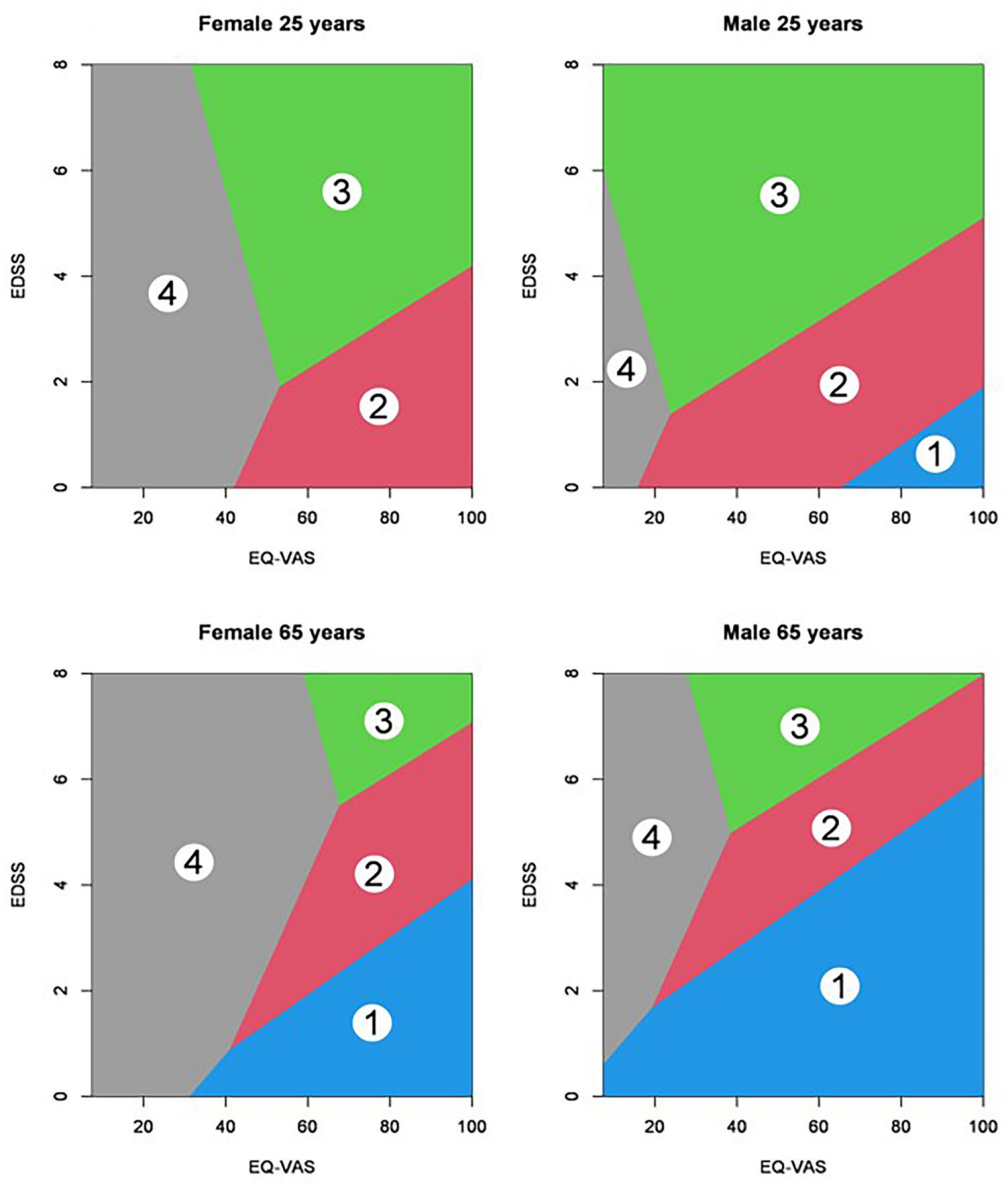
Visualization of areas with the highest probability of belonging to a certain segment on EDSS and EQ‐VAS scores, male and female participants at ages 25 and 65.

Probabilities for each point in the figure concerning EQ‐VAS and EDSS scores were calculated for men and women at ages 25 and 65 years, respectively. These probabilities are illustrated in Appendix S3 in curves representing a selection of probability levels. In comparison with Figure 1, these figures also include low probabilities.

In Figure 1, for women 25 years of age, there is a high probability for Segment 4 if the EQ‐VAS score is low, regardless of the EDSS score. With a high EQ‐VAS score, the probability is higher for Segment 3 if the EDSS score is high, while the probability is higher for Segment 2 if the EDSS score is low.

For women of 65 years, Segment 1 has the highest probability when the EQ‐VAS score is high and the EDSS score is high at the same time. The pattern of most probable for Segments 2 through 4 is pushed toward higher EDSS scores, meaning that now EDSS needs to be very high to make Segment 3 the most probable, while the EDSS score now needs to be medium to make Segment 2 the most probable (compared to women at age 25).

For men, the pattern looks roughly the same as for women, but it is pushed more to the left, meaning that a lower EQ‐VAS score is required to make Segment 4 the most probable (Figure 1).

In comparison, both men and women have higher probabilities of belonging to Segments 1 and 4 at age 65 than at age 25. Women at age 25 did not have the highest probability of belonging to Segment 1 at any point (Figure 1).

### 3.3 Segments and Deductive Content Analysis

#### 3.3.1 Perspectives on Life and Health

*Relations with others*: Close family and friends were the main sources of emotional, practical, and self‐care support across all segments. Integrity in choosing whom to share the diagnosis with was also important in every segment (Q1a, Table 7).

**TABLE 7:** Quotes from the qualitative data.

Q1 *a. (Segment 2) “My family knows about it—my children do, of course. My boss at work also knows because I sometimes need to take time off, but aside from my boss, I haven't discussed it with anyone at work. I plan to keep it quiet as long as nothing shows”* *b. (Segment 1)“ Generally, I am lucky and have tried to live as normally as possible”* *c. (Segment 4) “I have been affected mentally, like thinking about what things are going to look like in the future. How the disease will develop and such. It's tough. Maybe I have had the disease longer than I think, and what does that mean, will I get worse now … but I think as long as I can stand on my feet, I'll probably be happy.”*
Q2 *a. (Segment 3) “I exercise a lot. I walk frequently, and I've even bought an exercise machine for home use.”* *b. (Segment1) “Then as always, of course you're tired at times and so on, and I don't know, so to speak, whether it has to do with my medication or if it has to do with the disease, or if it's just like that in general, you can be tired in the spring and you can be tired in the fall and, well, a little bit like that”* *c. (Segment 4) “It has affected me to the point where I can no longer do the things I used to do. I can't cycle anymore; I can't do anything like that… I can't walk fast, and I can't walk for long either. When I go shopping, I have to stop because I can't walk far—it hurts, and those kinds of things are really frustrating.”* *d. (Segment 2) “I've had four different ones (medicines)—I'm currently on the fourth type. The other three were completely useless, so that's just how it goes sometimes; that's the journey, and we have to accept it. Of course, they start off with the ones that are probably the easiest and cheapest, naturally.”*
Q3 *a. (Segment 2) “Then I just got the diagnosis verdict right in my face, and nothing else happened. I got into my car—I remember it was completely dark—and I might as well have driven myself to death on the way home, because I almost started wondering if I should deliberately steer towards a truck or not…//…. It's an incredibly heavy, dreadful piece of news to receive, so you can't just let someone like that go, as it's really dangerous.”* *b. (Segment 1) “Most of my contacts are at the neurologist's and I have my annual visits and get in touch if I need to. It's great … // … But of course these doctor's appointments have been very fast … And then there have been these surveys and then maybe the computer has malfunctioned a bit and things like that, and so it has been a little quick in and quick out. So, of course, had I been able to exchange the 10 minutes for a conversation in peace and quiet where I, like, could have described my situation and my questions a little more vividly, that would have suited me better”* *c. (Segment 4)“ No, but the thing is, when you see that many others have it much worse than I do, it reminds me that not many people are as fortunate as I am. It almost makes me feel guilty for being so well off.”*
Q4 *a. (Segment 1) “In other words, when I need to get in touch, I manage to do so—and I'm not constantly being pestered; I don't find it bothersome either.”* *b. (Segment 4) “There is no coordination between the clinics, but sometimes they have talked to each other. Should it be like this? How long, then, until I will start to feel better, and what's the plan? A care plan would have been nice, I'm the kind of person who wants to know things. Well, should the treatment be changed, or should we continue like this, for how long?”* *c. (Segment 3) “First of all, the healthcare is already good. Secondly, it can always be improved—that's how I view it, starting with myself. I know I can always get better, even when I've hit a low point and couldn't do certain things; there's always room for improvement. Simply put, be receptive. You can educate yourself by reading up on things—and I do that too.”*

*Personal attitudes*: All persons described the importance of a positive attitude. In Segments 1 and 2, participants felt the disease had been “kind” and expressed a sense of fortune in life (Q1b, Table 7). In Segment 4, there was a sense of luck compared to worse diseases or situations.

General health was assessed on a scale from 1 (*very good*) to 5 (*very poor*). In Segment 1, two persons reported *very good* (1) and one *fairly good* (3) general health. In Segments 2 and 3, all rated their general health as *pretty good* (2). In Segment 4, ratings were *very good* (1), *fairly good* (3), and *poor* (4).

*Future perspectives*: In all segments, participants expressed being hopeful and positive for the future and being concerned about how symptoms could progress (Q1c, Table 7). Most persons were unaware of their prognosis. Worries and hopes were influenced by information from health care, their own thoughts on years with the diagnosis, and observing others with MS.

#### 3.3.2 Influence on Everyday Life

*Interests and activities*: Persons in all segments described engaging in physical exercise to promote health and also engaging in personal interests and social activities (Q2a, Table 7).

*Symptoms and consequences*: Symptoms across all segments included balancing difficulties, visual impairments, urinary incontinence, and fatigue. Differentiating MS symptoms from medication side effects, other conditions, or normal variations was a challenge they shared (Q2b, Table 7). Limited walking capacity was described in Segments 3 and 4.

In Segments 1 and 2, consequences ranged from none to minor difficulties with concentration, limited energy for social events, and issues with activities requiring good balance. Consequences in Segments 3 and 4 ranged from none to MS affecting many or all aspects of life (Q2c, Table 7). Sometimes other conditions also influenced the experienced consequences.

*Aspects of medication*: A shared experience was that finding the right medication was a difficult process (Q2d, Table 7). In Segment 1 all persons had medication. In Segments 3 and 4 most persons had drip (infusion) treatment ranging from monthly to every sixth month. Treatments for other conditions also existed. Both persons with and without medication expressed being satisfied with their current situation regarding medication.

#### 3.3.3 Relations With Health Care

*Diagnosis confirmation*: Experiences of shortages from healthcare in the diagnosis and confirmation process were described in all segments (Q3a, Table 7).

*One's individual requirements*: In Segment 1, contacts were mainly with the neurology unit, with no need for additional contacts, although some desired more personalized questions, information on medication, and joint planning in the contacts they had (Q3b, Table 7). In Segment 2, contact needs varied; some wanted fewer contacts due to unnecessary testing, while others desired more continuity in contacts with their neurologist. In Segment 4, other medical conditions influenced contact needs over time. All segments described examinations with an MRI as part of their contacts.

*Building knowledge*: In all segments, searching the internet was the most common way of learning about MS in the initial phase, and they addressed both strengths and limitations with online information. Few referred to healthcare as a resource. Learning from others with MS varied within the segments rather than between them; some found it helpful for knowledge building, while others saw it as burdensome and leading to a negative view of the disease (Q3c, Table 7).

#### 3.3.4 Shared Healthcare Processes

*Access to care*: All segments generally described good access to the neurology unit (Q4b, Table 7). In Segment 2, primary care sometimes was seen as a barrier to getting specialized care. In Segments 3 and 4, some wanted to be able to choose video consultations with physicians to avoid energy costs and the burden of travelling. Access was limited by restricted telephone hours.

*Planning and coordination*: Having a care plan was seen as beneficial in all segments. In Segment 1, this could include examinations and planned consultations for the coming year. In Segments 3 and 4, coordination between and within clinics together with a timeline for interventions such as medications were seen as important components (Q4b, Table 7).

*General perceptions*: Overall, healthcare was perceived positively across all segments. Even in cases of negative encounters, the general view of healthcare remained positive, with negative events often being seen as isolated incidents (Q4c, Table 7).

### 3.4 Merged Interpretation

The joint display of qualitative and quantitative data is presented in Table 8. The eight themes reflect integrated perspectives from both types of data. The theme “Characteristics of the population” include data on age, gender, and type of MS. “Disability and function” include quantitative data on EDSS and the 6‐min WT, as well as qualitative aspects of disability and function from the qualitative theme Influence on everyday life. “Symptoms and consequences” include findings from the variables “MS‐kollen” and MSIS, along with related findings from Influence on everyday life. “Fatigue and cognition” include quantitative data from FMSC and SDMT, combined with related findings from the qualitative theme, Influence on everyday life. “Perspectives on health” includes quantitative data from EQ‐VAS and qualitative perspectives from the theme perspectives on life and health. “Examinations” includes the quantitative variables MRI and relapses, together with related qualitative content from relations with healthcare. “Interactions with healthcare” include quantitative data on contact frequencies and qualitative data on experiences of healthcare contacts from relations with healthcare and shared healthcare processes. “Disease duration and the future” include quantitative data on disease duration and qualitative findings from perspectives on life and health. Finally, elements that could not be integrated are gathered under “Discord in data.”

**TABLE 8:** Joint display of quantitative data and qualitative data with merged interpretation.

Theme	Merged interpretation
Characteristics of the population	There were more women than men across all segments. Mean age varied between 46 and 50 years. There were higher probability of belonging to Segments 1 and 4 at age 65 than at 25. In the qualitative data, the person's own age sometimes influenced their views and plans for the future. The qualitative findings expand on age as influencing perspectives of hope, fears, and planning for the future.
Disability and function	EDSS scores (Figure 1) increased, and 6‐min walk test distance decreased from Segments 1 to 4. Higher EDSS increased the probability of belonging to Segment 3 but had less influence on Segment 4. Qualitative findings confirmed quantitative findings on mobility limitations in Segments 3 and 4. Variability in Segment 4 was that some had no limitations, while others experienced severe mobility restrictions. The qualitative data provide additional context for the variability in Segment 4, indicating that not all individuals experience severe mobility issues.
Symptoms and consequences	Increased symptoms (Figure 2) and consequences with more frequent healthcare contacts were found. The qualitative data confirms the overall trend of increasing symptoms but highlights individual differences in how symptoms are perceived. Expanding findings were difficulties of differentiating MS symptoms from other conditions.
Fatigue and cognition	Quantitative data describe increased fatigue (Figure 3) and cognitive symptoms with greater healthcare contact frequency. Qualitative findings describe more diverse patterns within and between segments. Fatigue had varying impacts on social, physical, and cognitive aspects of life. Difficulties differentiating fatigue from general tiredness (Segment 1) and depression (Segment 4) existed. In Segment 2, no fatigue or cognitive symptoms were expressed. The qualitative findings expand on the quantitative, illustrating that the impact of fatigue varies between individuals.
Perspectives on health	EQ‐VAS (Figure 4) and general health assessments declined from Segments 1 to 4. High EQ‐VAS scores were associated with Segment 1, while low scores were linked to Segment 4. A positive attitude was important across all segments. In Segments 1 and 2, individuals felt “lucky” their disease was mild. In Segment 4, feeling “lucky” was framed as not having a worse condition. The qualitative data provides insight into how individuals interpret their health, showing that perceived well‐being is influenced by personal perspectives and attitudes.
Examinations	MRI and relapse variables contained outliers and extreme values across segments. MRI was a recurring healthcare event for all persons. MRI results, on the other hand, were not described in the qualitative data. Relapses were briefly mentioned concerning transitory symptoms and medication effects.
Interactions with healthcare	The different segments represented different contact frequencies in health care (Figure 5). Qualitative data expanded on the content and needs of contacts. Segment 1: Primarily neurology unit, no additional contact requests but preferred personalized content. Segment 2: Varied preferences—some needed more contacts, others wanted fewer. Segment 4: Other medical conditions influenced contact needs over time.
Disease duration and the future	Some individuals considered the disease timeline in relation to prognosis and most lacked awareness of their long‐term prognosis. Future concerns were shaped by healthcare information, personal reflections, and observations of others with MS. While the quantitative data found no significant differences in disease duration (Figure 6), the qualitative findings suggest that individuals' awareness and expectations for the future differed and for some, this were related to disease duration.
Discord in data	Qualitative themes included relationships, medication aspects, and knowledge‐building, which were absent from the quantitative data. The qualitative findings illustrate that while type of MS is present in the quantitative data, most of the participants did not know what type they had, highlighting differences in focus between the datasets.

**FIGURE 1:**
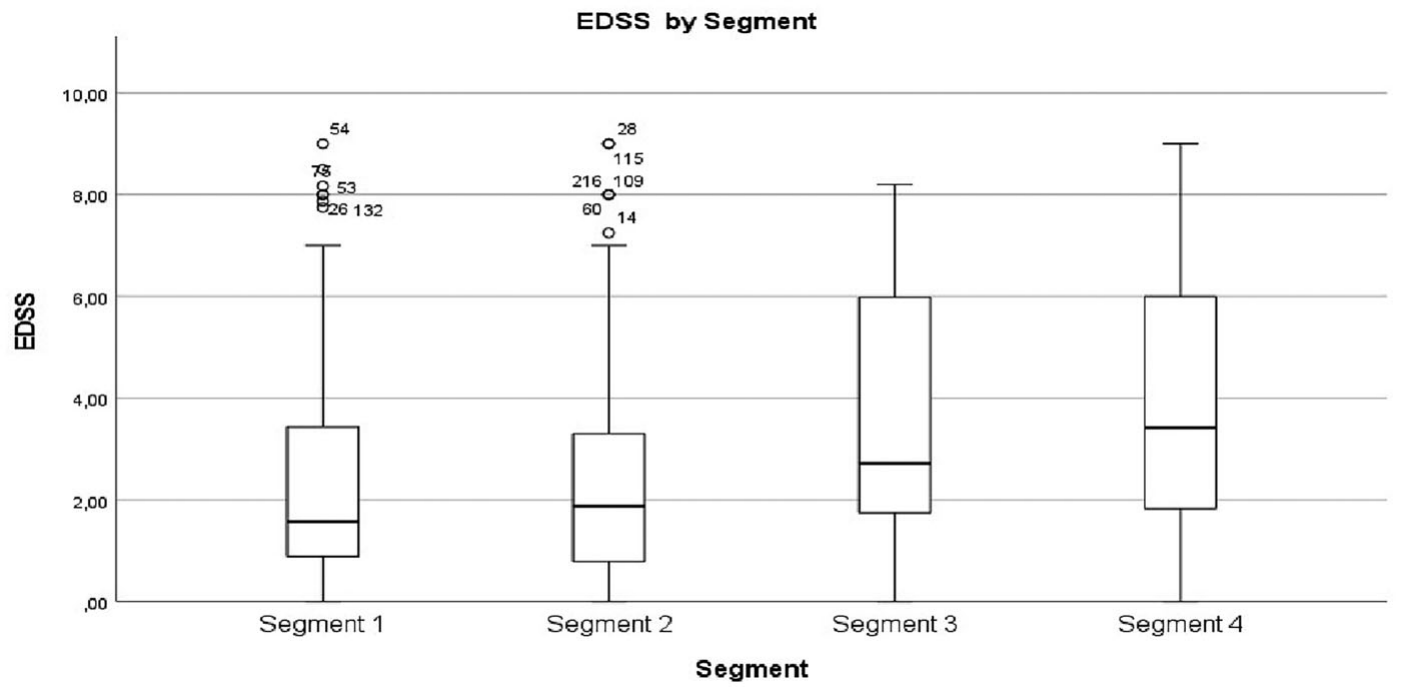
Visualization of mean EDSS score by segment.

**FIGURE 2:**
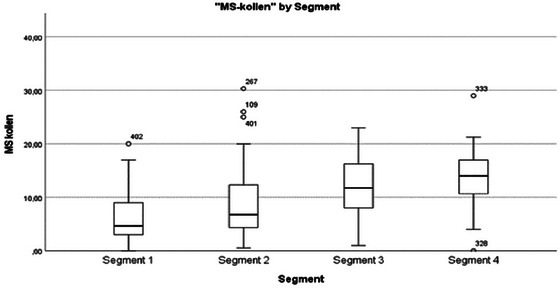
“Ms kollen” by segment

**FIGURE 3:**
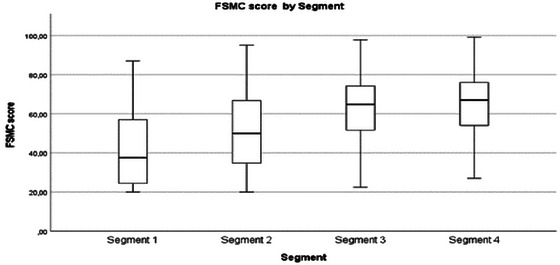
FSMC score by segment

**FIGURE 4:**
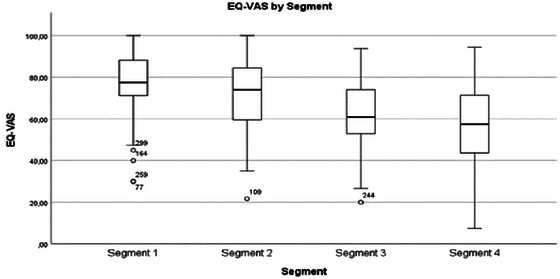
EQ‐VAS by segment

**FIGURE 5:**
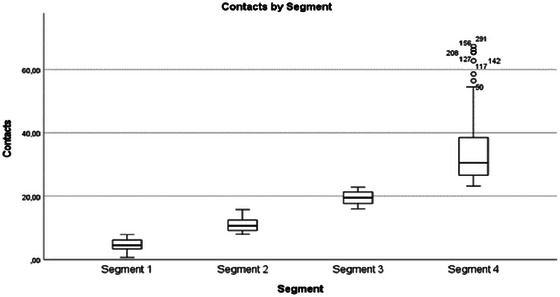
Healthcare contacts by segment

**FIGURE 6:**
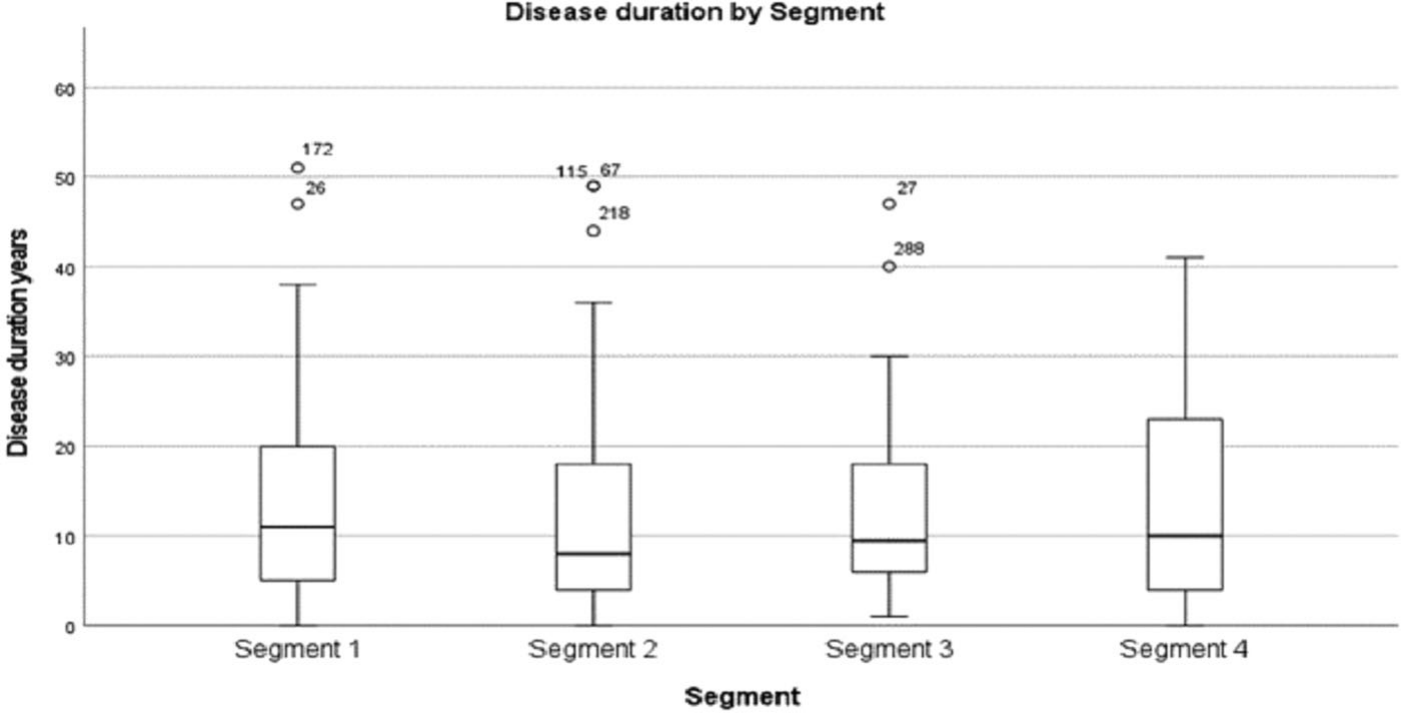
Disease duration by segment

## 4 Discussion

The more general findings in our study on distribution of variables and experiences across segments, with increase in symptoms, loss of function, and deterioration of health experience correlating with increased levels of contact frequency from Segments 1 to 4 and the influence of comorbidities has also been found in other studies (Berrigan et al. 2016; Marrie 2017, 2019; Marrie et al. 2015). The quantitative regression results add another perspective; we go from the distribution of variables between segments to how the distribution between segments is affected by the four explanatory variables (age, gender, EQ‐VAS, and EDSS). These data provide insights into what influences belonging to a certain segment and challenge the “linear thinking” of each variable. For example, low EQ‐VAS scores increase the probability of belonging to the segment with most contacts, whereas EDSS (function/symptoms) scores need to be very high to make the segment with the second most contacts the most probable. So, at the same time as we know from variable data that the distribution of EDSS scores as an individual variable increases from Segments 1 through 4, we learned from the regression that high EDSS scores affect probabilities more in Segment 3 than in Segment 4. These findings suggest that healthcare utilization is complex rather than linear (Ng et al. 2024) and the relationship between EDSS and healthcare contacts may be nonlinear, potentially exhibiting a threshold beyond which increases in EDSS do not lead to changes in contact frequency. For example, one study on EDSS and EQ‐5D‐5L found a correlation between EDSS and quality of life up to an EDSS score of 6; beyond that, there was significantly greater variability in EQ‐5D‐5L scores (Fogarty et al. 2013). This could imply that at higher levels of disability health‐related quality of life becomes more inconsistent, and the variables play different roles for healthcare utilization. Further exploration on if, and how these predictors are of importance are suggested, especially for segments having high level of contact frequencies in healthcare. In addition, since EQ‐VAS is a self‐reported measure, low scores might have a greater impact on healthcare‐seeking behavior, while EDSS reflects a clinical assessment.

Relations with others, aspects of medication, and knowledge building were areas identified as discord in data (Table 8). These qualitative subthemes all relate to aspects of self‐care and might suggest that this is overlooked by health care, but of importance to patients. This discord can be viewed in relation to previous research that identified differences between patients' and professionals' views on self‐care (Riggare 2022), illness (Gaille 2018), and patient participation (Eldh et al. 2010). This study underlines the need for more integrated and coproduced care and self‐care approaches for persons with MS, just as others have before (Bartolomeu Pires et al. 2024; Docteur and Coulter 2012; Marrie 2017, 2019). In the Swedish context, where inadequate coordination of care among healthcare providers has been described (Docteur and Coulter 2012), these findings and our approach to exploring contact frequencies, including both primary care and specialized hospital care for individuals with chronic disease, can be valuable. They offer insights into how a more holistic approach to care coordination might be achieved, ultimately reducing the overall burden of illness. Notably, a previous study (Lind et al. 2022) found that most healthcare contacts for the MS population occur in primary care rather than in specialized hospital care.

### 4.1 Strengths and Limitations

The variation in the number of persons included in different quantitative variables affects the likelihood of sampling errors and the representation of the population. The variables have strengths and weaknesses in what they capture and in their reliability and validity (Benedict et al. 2017; Ernstsson et al. 2017; Hawton et al. 2012; Hobart et al. 2001; Hoogervorst et al. 2004; Meyer‐Moock et al. 2014; Penner et al. 2009; Sandry et al. 2021; Sharrack and Hughes 1999; Teni et al. 2023; Wetzel et al. 2011). Using statistical tests for differences between segments allows for understanding the effects of group membership on that particular variable but runs the risk of a Type I error. Separate analyses do not account for potential interactions or correlations between variables and might overlook important relationships in the data (Harrison et al. 2021). The multinomial regression with simultaneous examination of multiple explanatory variables provides a holistic understanding of the relationships between variables and segments (Allison 2012). The qualitative sampling approach resulted in 2–3 persons within each segment, which can influence the generalizability and knowledge claim of this study and the findings must be viewed in relation to this. The discussion on findings in relation to other studies can, on the other hand strengthen aspects of generalizability.

Mixed‐methods studies often involve a larger quantitative sample alongside a smaller qualitative one. In our study quantitative data offers broader pattern detection, but it may miss individual nuances. In contrast, the qualitative data provides in‐depth contextual insights, although the smaller, purposively selected sample limits generalizability. The knowledge claim of this study is not to give a complete picture but rather to explore different perspectives that might influence segments representing different contact levels with health care in a population of persons living with MS.

The timeframe for this study was due to accessible data from the administrative systems. Context factors that could influence data discussed were for example organizational changes, COVID‐19, national trends, development of MS care, and staffing fluctuations study. When looking at contacts from 2013 (*n* = 3748) to 2021 (*n* = 4288), there was a general increase of contacts over time. In relation to COVID‐19 there was a decline. The year with most contacts was 2018 (*n* = 5101). Other studies have described the impact of COVID‐19 on healthcare contacts and interruptions in MS care (Vogel et al. 2020) as well as reduction in occupational engagement and social and daily activities which can impact perceived quality of life and function (Goverover et al. 2022). Impact of COVID‐19 on contact variables and qualitative data most likely exist.

In this explorative study, mean values were used. A mixed‐effects model would capture individual variation over time and could serve as an important next step in exploring healthcare contacts.

### 4.2 Implications for Policy, Practice, and Research

The findings can be used to improve care in general as well as address specific needs and requirements in the different contact levels. Healthcare contacts and interventions would likely need to be designed differently depending on both individual factors and level of contact frequency. Increased awareness of differences regarding the probability of segment belonging, along with an understanding of the impact of comorbidity, can be important in the coproduction of care that creates the most value for both the individual and the healthcare system. The more general improvement areas identified regarded the content of healthcare contacts, such as self‐care and more person‐centered approaches.

We suggest to further explore the segment‐specific needs in relation to the explanatory variables identified in this study, together with the knowledge of persons living with MS and professionals. In future research, socioeconomic factors would also be valuable to explore in relation to healthcare utilization (Agerholm et al. 2013).

## 5 Conclusions

Our findings suggest that persons with the most healthcare contacts in general have more symptoms, loss of function, and poorer overall health compared to those with fewer contacts. The probability of having a certain level of contact frequency is influenced by the explanatory variables age, gender, general health (EQ‐VAS), and disability status (EDSS), with the different variables playing different roles for each level. The qualitative findings describe different experiences within the different segments and the influence of comorbidities. The data integration adds to the exploration through confirmation, expansion, and discord regarding for example personal context and aspects of self‐care that may be overlooked in healthcare contacts.

## Author Contributions

**Sofia Persson**: writing – original draft, writing – review and editing, methodology, formal analysis, visualization, data curation, validation. **Ann‐Christine Andersson**: formal analysis, data curation, supervision, writing – review and editing, methodology, validation. **Boel Andersson Gäre**: supervision, formal analysis, writing – review and editing, methodology, validation. **Jonas Lind**: supervision, data curation, formal analysis, writing – review and editing, methodology, validation, visualization.

## Conflicts of Interest

The authors declare no conflicts of interest.

## Peer Review

The peer review history for this article is available at https://publons.com/publon/10.1002/brb3.70634

## Supporting information




**Supporting Appendix**: brb370634‐sup‐0001‐Appendix1.docx

**Supporting Appendix**: brb370634‐sup‐0002‐Appendix2.docx

**Supporting Appendix**: brb370634‐sup‐0003‐Appendix3.docx

**Supporting Appendix**: brb370634‐sup‐0004‐Appendix4.docx

## Data Availability

In addition to the data provided in the appendix, data supporting the findings of this study are available from the corresponding author upon reasonable request.
