# Supplementary material for: Exploring Different Levels of Contact Frequency in Multiple Sclerosis Care
Source: Brain Behav. 2025 Jul 7;15(7):e70634. doi: 10.1002/brb3.70634 (PMC12230343; doi:10.1002/brb3.70634)
Supplement: Supplementary file 1 — Supporting Appendix: brb370634‐sup‐0001‐Appendix1.docx [file BRB3-15-e70634-s001.docx]

# Appendix 1: Variables from the MS Registry

Table 1 Overview of variables explored in regards to reporting, scales, what is measured, items and scores and outcomes

| Variable | Reporting | Scales | Area measured | Items and scores | Results and reference values |
| --- | --- | --- | --- | --- | --- |
| Expanded Disability Status Scale (EDSS) | Clinician-administered assessment scale | Ordinal rating system from 0 (= normal neurological status) through 10 (=death due to MS) | Evaluating the functional systems of the central nervous system | Scores are in half unit steps – 3, 3.5, 4 and so on. | The greater the level of disability, the higher the score out of ten. |
| MS Impact scale (MSIS 29) | Patient reported | Likert scale: 1 -5 (1=not at all, through 5=incredibly much) | Effects of MS latest two weeks | MSIS Physical, 20 items, score between min 20, max 100 | Total score is derived by summing items and transforming them into a score out of 100; higher scores imply a greater degree of disability. |
|  |  |  |  | MSIS Psychological 9 items, score min 9, max 45 |  |
| Fatigue Scale for Motor and Cognitive functions (FSMC) | Patient reported | Likert scale:1–5 (1=does not apply at all, through 5= applies completely) | Grading of fatigue | 10 Motor questions | A sum score of ≥43 is categorized as mild fatigue, ≥53 as moderate fatigue and ≥63 as severe fatigue |
|  |  |  |  | 10 Cognitive questions |  |
|  |  |  |  | The total possible score ranges from 20–100 points |  |
| Symbol Digit Modalities Test (SDMT) | Test conducted in clinic | Summing the number of correct substitutions within the 90 second interval | General assessment of cognition | Max = 110 | Low values can indicate cognitive difficulties |
| EQ- VAS General health | Patient reported | The Visual Analog Scale (VAS), perceived health status from 0 = worst imaginable health status through 100 = best imaginable health status. | Patient's perceived overall health | Max= 100 | The higher the rating, the better the perceived health |
| MS-kollen (Swedish version of The Guy's Neurological Disability Scale) | Patient reported | Likert scale from 0= no symptoms 1 through 3 = severe, disabling symptoms | Symptom assessment | Max score 56 | Higher scores indicate more symptoms |
| 6 minutes Walking test (6min WT) | Test conducted in clinic | Distance walked during 6 minutes | Gait function, endurance and aerobic exercise capacity. | In healthy subjects, the 6-min walk distance (6MWD) ranges from 400 to 700 m, | Low score correlates with lower function |
| Relapses | Reported by clinician | Number of relapses | Disease activity | Annual number of reported relapses | Low scores indicates low disease activity |
| New T2 enhancing lesions on magnetic resonance imaging (MRI) | The overall number of MS lesions in the brain | Number of lesions | Disease activity | Annual number of new T2 enhancing lesions | Low scores indicates low disease activity |
