# Supplementary material for: Exploring Different Levels of Contact Frequency in Multiple Sclerosis Care
Source: Brain Behav. 2025 Jul 7;15(7):e70634. doi: 10.1002/brb3.70634 (PMC12230343; doi:10.1002/brb3.70634)
Supplement: Supplementary file 2 — Supporting Appendix: brb370634‐sup‐0002‐Appendix2.docx [file BRB3-15-e70634-s004.docx]

## Appendix 2. Qualitative framework and example of analysis

Deductive Framework (table 1) and Analysis example (table 2)

*Table 1. Deductive framework granted from thematic analysis on lived experiences (1)*

| **Themes** | **Subthemes** |
| --- | --- |
| A. Perspectives on life and health | Relations with others, Personal attitudes, Future perspectives |
| B. Influence on everyday life | Interests and activities, Symptoms and consequences, Aspects of medication |
| C. Relations with healthcare | Diagnosis confirmation, One's individual requirements, Building knowledge |
| D. Shared healthcare processes | Access to care, Planning and coordination, General perceptions |

*Table 2. Example of the deductive analysis with themes, subthemes, codes and open codes based on segments*

| **Theme** | **Subthemes** | **Codes** | *Segment 1,*  *Data extract* | *Segment 2, Data extract* | *Segment 3, Data extract* | *Segment 4, Data extract* |
| --- | --- | --- | --- | --- | --- | --- |
| Shared healthcare processes | Access to care | Organizational pathways  Practical context  Personal requirements | *A.*  *"No, I call that MS nurse if I need any contact or help. It works."*  *B.*  *"As soon as I’ve called in, as soon as I’ve said something, I’ve gotten a response or feedback right away. It feels like there’s time for me when I call, so it feels good."*  *C.*  *"I’ve called the neurology clinic with questions. The care has been supportive and has been there for me."* | *D. "Once you get past primary care, it’s absolutely fantastic. When you finally reach out, it has usually gone quite far, so you want something to happen. I don’t see healthcare regularly in any way. I can’t drive because of my vision, my wife has to drive."*  *E.”The interaction between the hospital and primary care doesn’t work very well."* | *F. "I'm not the type to complain right away, so when I do reach out, the doctor usually says, 'If you say it’s bad, then it is, so I want to see you.' The accessibility is good now that I’ve gotten the diagnosis, but before, it wasn’t as good. I have treatment once a month, so I’m in contact with healthcare then. It’s easy to get in touch with the clinic if there’s anything. But there are certain phone hours, so sometimes you have to wait a bit."*  *G."I can call the medical department whenever I feel like it, exchange a few words with them, and see what I should do."* | *H. "You always get an appointment when you need it."*  *I. "It’s difficult to get to the clinic. I would have needed a parking permit, but I don’t get one. This makes me dependent on getting a ride to and from healthcare visits."*  *J."It would have been good to have more video calls... some days you’re not feeling well... If you have any questions, you get answers right away. It feels like the person knows who you are when you call. That feels reassuring."* |

1. Persson S, Andersson AC, Gäre BA, Lindenfalk B, Lind J. Lived experience of persons with multiple sclerosis: A qualitative interview study. Brain Behav. 2023;13(7):e3104.
