# Supplementary material for: Exploring Different Levels of Contact Frequency in Multiple Sclerosis Care
Source: Brain Behav. 2025 Jul 7;15(7):e70634. doi: 10.1002/brb3.70634 (PMC12230343; doi:10.1002/brb3.70634)
Supplement: Supplementary file 3 — Supporting Appendix: brb370634‐sup‐0003‐Appendix3.docx [file BRB3-15-e70634-s003.docx]

## Appendix 3. Statistical tests differences between segments and visualization of multinomial regression

Differences in each of the included variables across segments were analyzed using graphs (appendix 4) and statistical tests: one-way between-subjects analysis of variance (ANOVA) for normally distributed data (Table 1), the Kruskal-Wallis test when the normality assumption was not met (Table 2), and the chi-square test for categorical data (Table 3). Effect sizes were calculated using eta squared (η²) for ANOVA, Phi for chi-square tests, and epsilon squared (ε²) for Kruskal-Wallis (1, 2).

Further visualizations of the multinomial regression are presented with curves of probabilities at the same probability level in each segment for men and female participants at age 25 on different EDSS and EQ-VAS scores (figure 1a) and curves of probabilities at the same probability level in each segment for male and female participants at age 65 on different EDSS and EQ-VAS scores (figure 1b).

*Table 1. One way between subjects ANOVA for normally distributed variables*

| **Variable** | **F(df, N)** | **P-value** | **η2* including CI 95%** |
| --- | --- | --- | --- |
| Age | F(3, 401) = 2.399 | 0.067 | 0.018,  [0.000, 0.045] |
| SDMT | F(3, 228) = 3.651 | 0.013 | 0.046,  [0.002, 0.099] |

* Small 0. 01, medium 0.06, and large 0.14.

*Table 2. Kruskal Wallis for non-parametric variables*

| **Variable** | **X^2^ (df, N)** | **P-value** | **ε² ***** | **Bootstrap-based 95% confidence intervals** |
| --- | --- | --- | --- | --- |
| EDSS | (3, 339)= 26.907* | < 0.001 | 0.079 | Segment 1:[1.9155, 2.7488], Segment 2:[2.0525, 2.9336]), Segment 3: [2.9268, 4.1658], Segment 4: [3.3201, 4.5874] |
| FSMC motor | (3, 153) = 27.560 | < 0.001 | 0.18 | Segment 1: [1,9155, 2,7488 ]  Segment 2:[2.0525, 2.9336]  Segment 3: [2.9268, 4.1658]  Segment 4: [3.3201, 4.5874] |
| FSMC cog | (3,153) = 21.352 | < 0.001 | 0.14 | Segment 1:[17.89, 23.95  Segment 2: 22.88, 28.78  Segment 3: [26.61, 34.03]  Segment 4: [28.21, 35.24] |
| T2 | Graphic presentation** |  |  | Segment 1: [1.0097, 1.9806]  Segment 2: 1.3636, 3.2645]  Segment 3: [0.5850, 2.1803]  Segment 4: [1.0944, 3.1376] |
| Relapses | Graphic presentation** |  |  | Segment 1: [0.3899, 0.7087]  Segment 2: [0.4286, 0.7916]  Segment 3: [0.4203, 0.9818]  Segment 4: [0.2858, 0.7068] |
| MSIS phys | (3, 204) = 47.800 | <0.001 | 0.23 | Segment 1: [8.10, 13.26]  Segment 2: [18.51, 29.51]  Segment 3: [25.62, 38.98]  Segment 4: [30.53, 42.77] |
| MSIS psych | (3, 204) = 41.576 | <0.001 | 0.204 | Segment 1: [13.94, 21.81]  Segment 2: [27.05, 38.86]  Segment 3: [29.97, 42.38]  Segment 4: [38.55, 51.52] |
| “MS-kollen” | (3, 160) = 29.242 | < 0.001 | 0.18 | Segment 1: [4.93, 7.95]  Segment 2: [7.14, 10.83]  Segment 3: [9.83, 13.87]  Segment 4: [11.39, 15.66] |
| 6 min WT | Graphic presentation ** |  |  | Segment 1: [540.08, 614.76]  Segment 2: [552.75, 604.16]  Segment 3: [423.43, 528.72]  Segment 4: [373.43, 488.34] |
| EQ-VAS | (3, 241) =39.230 | <0.001 | 0.16 | Segment 1: [72.83, 79.85]  Segment 2: [67.46, 74.76]  Segment 3: [56.52, 66.93]  Segment 4: [51.44, 62.17] |
| Dis duration | (3, 405) =2.916 | <0.405 | 0.007 | Segment 1: [11.60, 15.07]  Segment 2: [9.94, 13.72]  Segment 3: [10.17, 14.76]  Segment 4: [10.46, 15.69] |

*Outliers in data (appendix 4)

** Outliers and extreme values, only graphical illustration (appendix 4)

** *Small 0, 01, medium 0.06, and large 0, 14 or higher

*Table 3. Chi- Square test for categorical variables*

| **Variable** | **X^2^ (df, N)** |  | **P-value** | **Phi***** |
| --- | --- | --- | --- | --- |
| Gender | (3, 405) = 8.893 |  | <0.001 | 0.148 |
| Type of MS | (6, 350) = 25.086 |  | <0.001 | 0.268 |

***Weak around 0.1, Medium around 0.3, Strong around 0.5 or higher


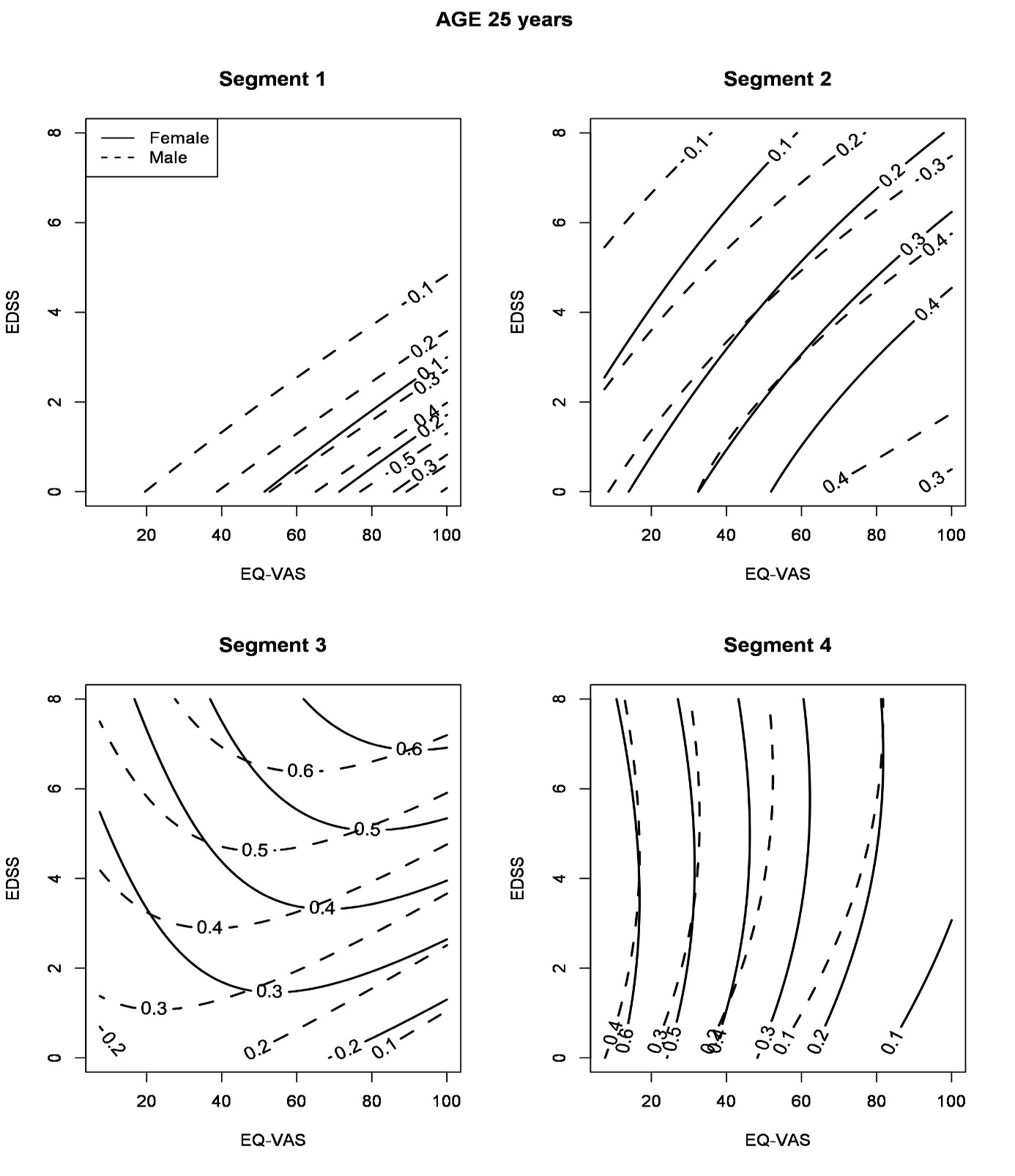


*Figure 1a. Curves of probabilities at the same probability level in each segment for men and female participants at age 25 on different EDSS and EQ-VAS scores*

*
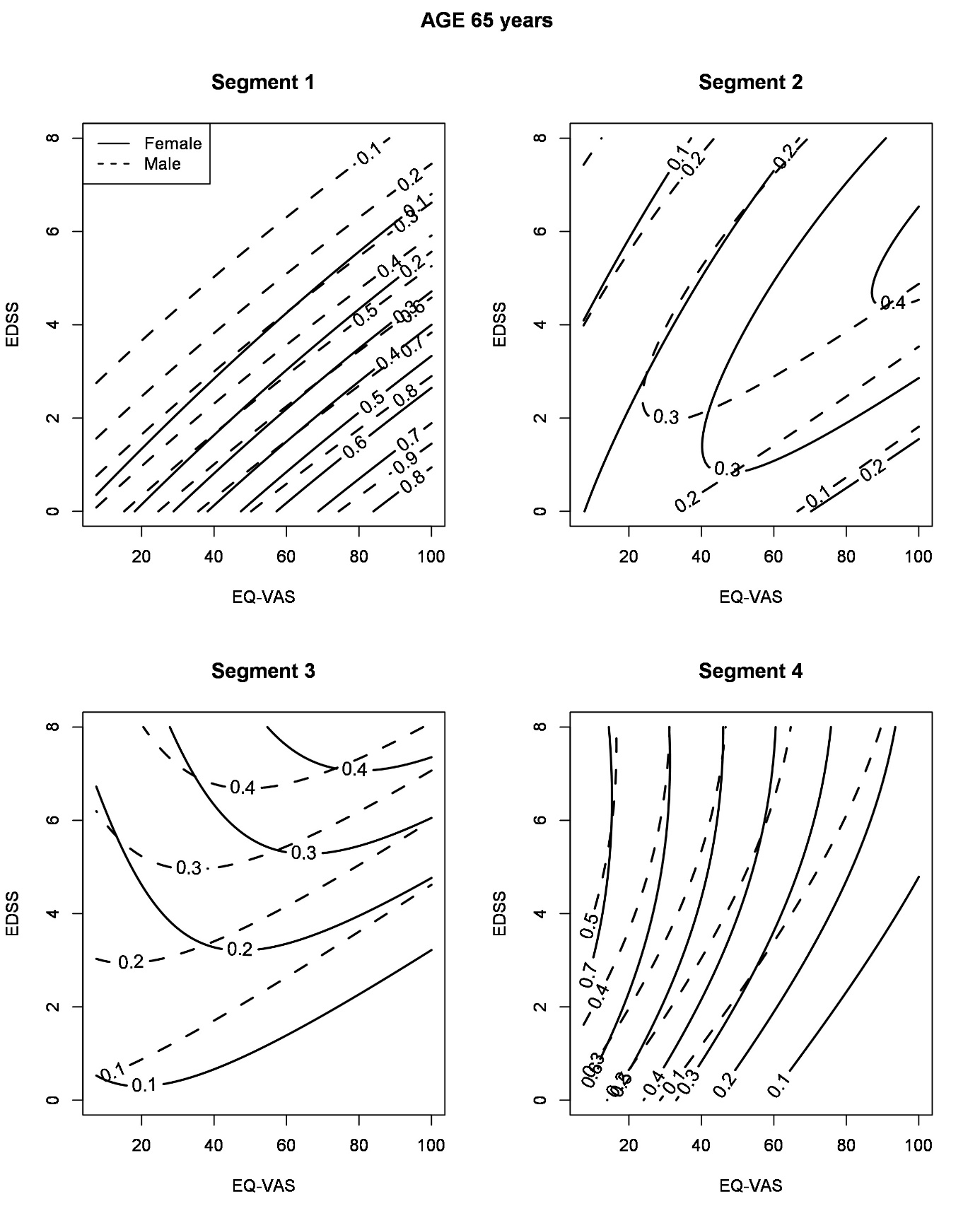
*

*Figure 1b. Curves of probabilities at the same probability level in each segment for male and female participants at age 65 on different EDSS and EQ-VAS scores*

References:

1. Harrison V, Kemp R, Brace N, Snelgar R. SPSS for psychologists. Seventh edition ed. London: Macmillan International Higher Education; 2021.

2. Fritz CO, Morris PE, Richler JJ. Effect size estimates: Current use, calculations, and interpretation. Journal of experimental psychology General. 2012;141(1):2-18.
