# Supplementary material for: Exploring Different Levels of Contact Frequency in Multiple Sclerosis Care
Source: Brain Behav. 2025 Jul 7;15(7):e70634. doi: 10.1002/brb3.70634 (PMC12230343; doi:10.1002/brb3.70634)

## Appendix 4 QUAN Graphs

Presentation of simple boxplots for variables explored in the study. Age (figure 1), EDSS (figure 2) , FSMC cog (figure 3), FSMC motor (figure 4), MRI new T2 (figure 5), MSIS Phys (figure 6), MSIS Psych (figure 7), Relapses (figure 8), MS-kollen (figure 9), SDMT (figure 10), 6 min WT (figure 11), EQ-VAS (figure 12), disease duration (figure 13) and bar charts for Gender (figure 14) and type of MS (figure 15).

Figure 1. Simple box plot of age by segment


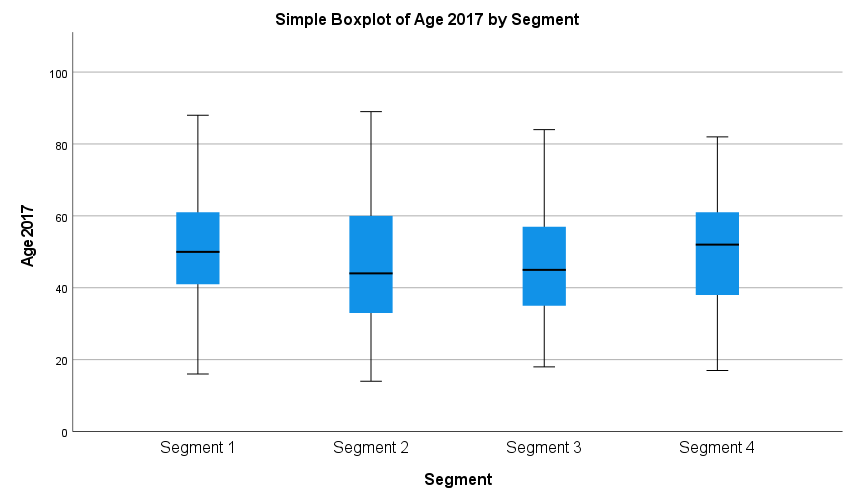


Figure 2. Simple boxplot of EDSS by segment


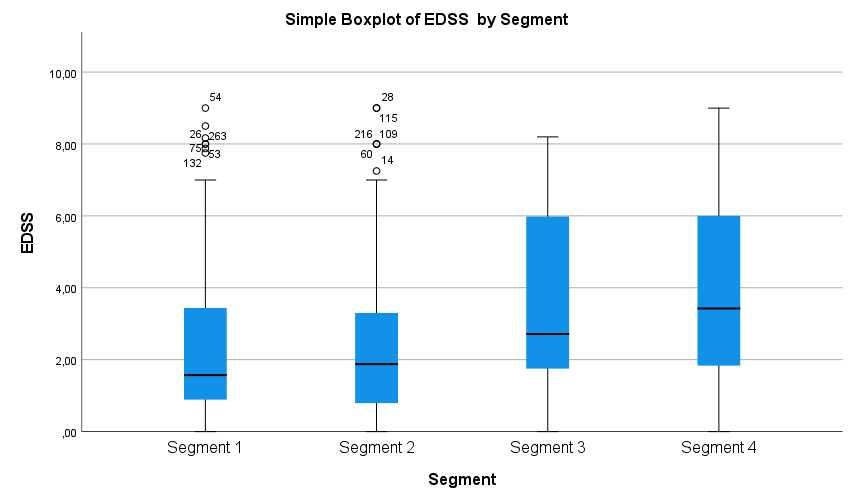


Figure 3. Simple boxplot of FSMC cogn. by segment


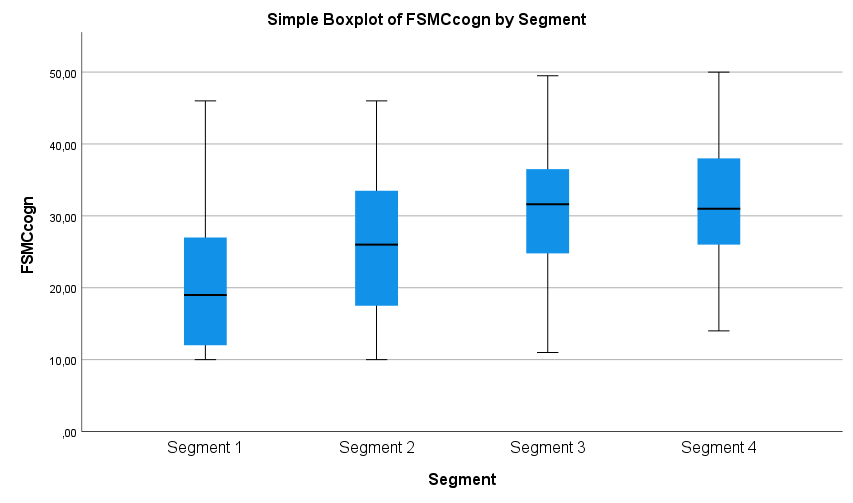


Figure 4. Simple boxplot of FSMC motor by segment


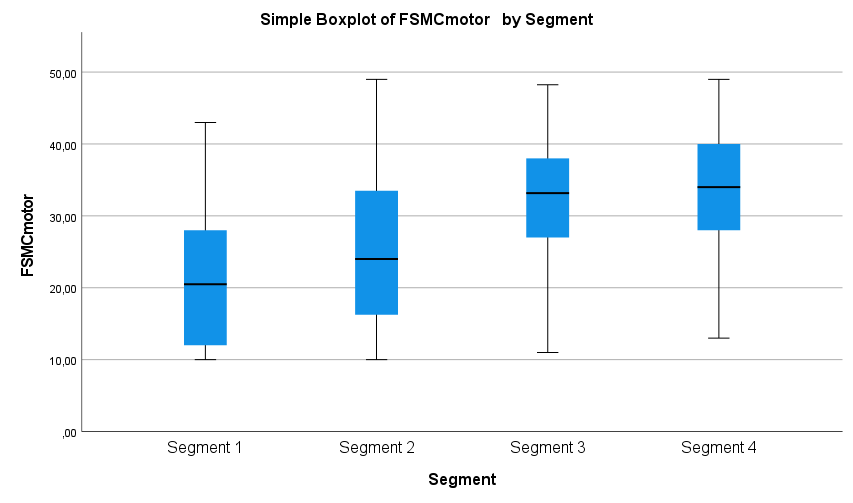


Figure 5. Simple boxplot of MRI T2 by segment


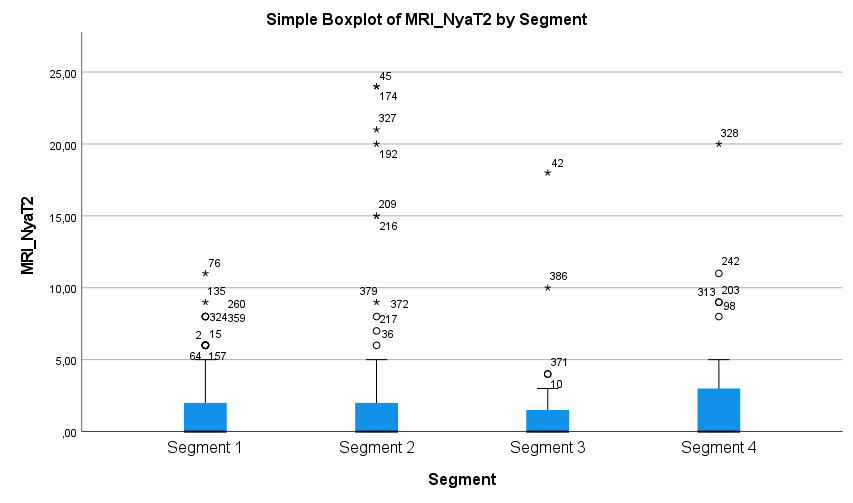


Figure 6. Simple boxplot of MSIS Psych by segment


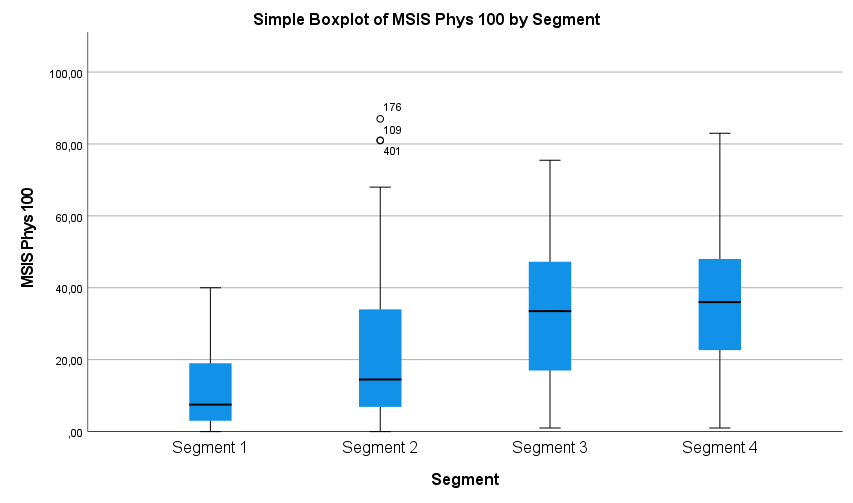


Figure 7. Simple boxplot of MSIS psych by segment


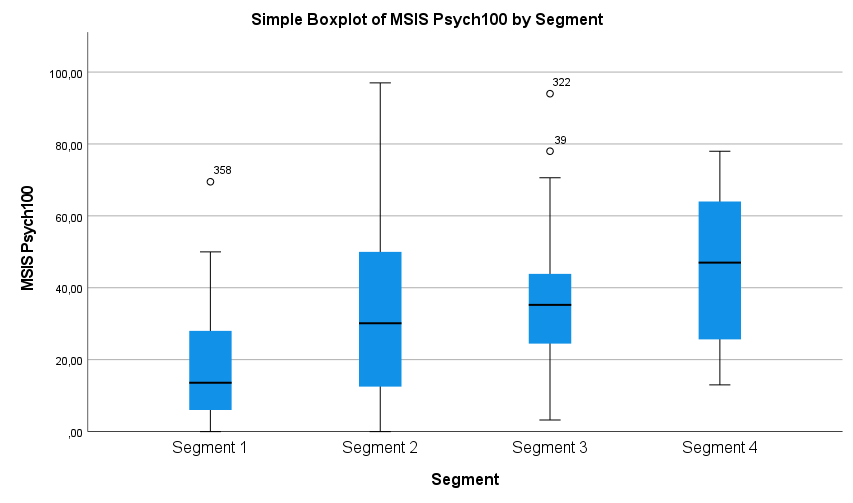


Figure 8. Simple boxplot of relapses by segment


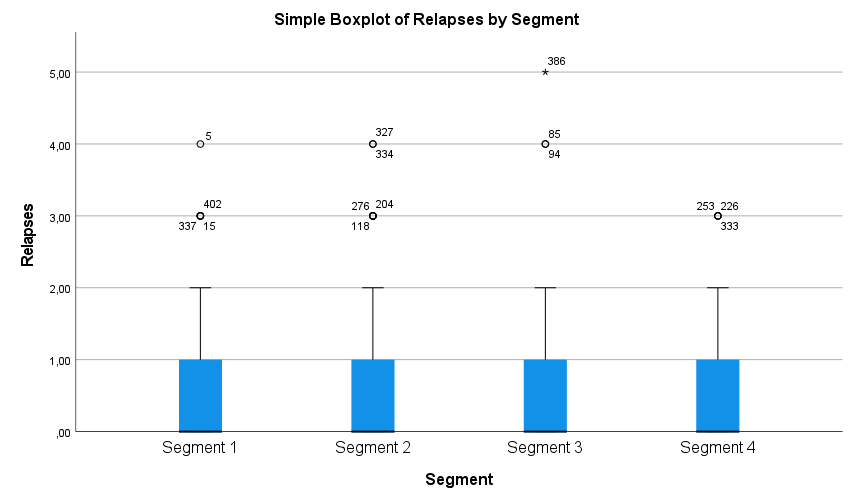


Figure 9 Simple boxplot of MS-kollen by segment


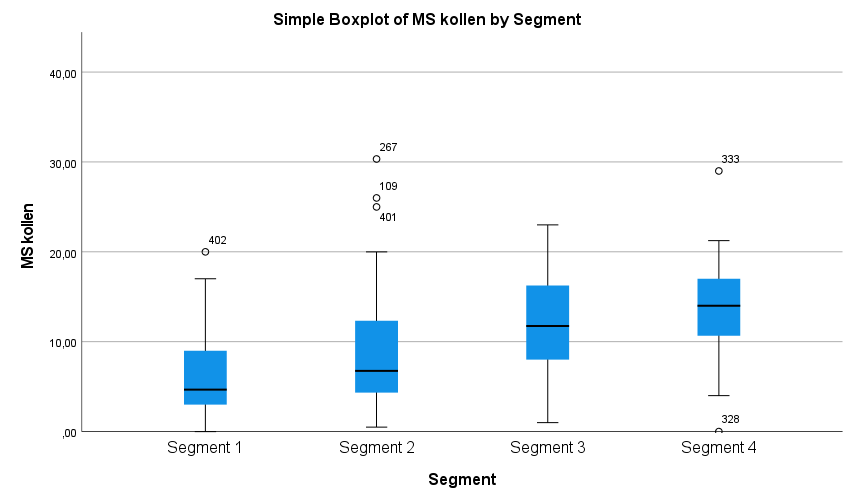


Figure 10. Simple boxplot of SDMT by segment


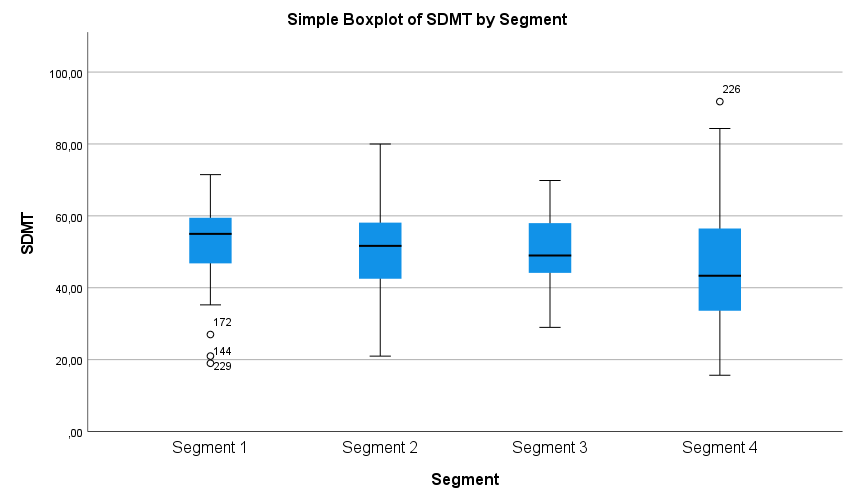


Figure 11. Simple boxplot of 6 min WT by segment


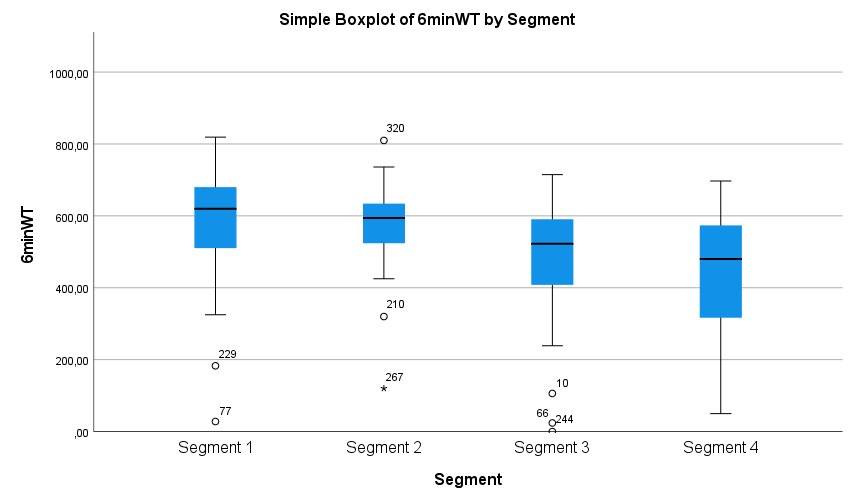


Figure 12. Simple boxplot of EQ-VAS by segment


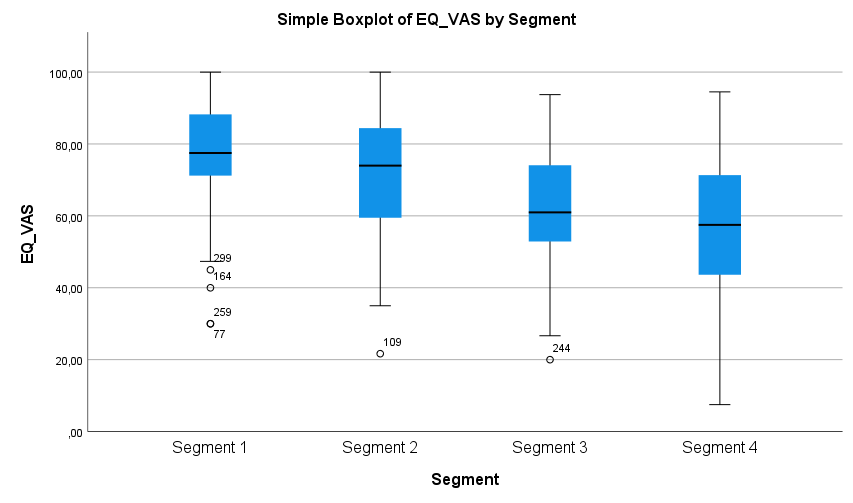


Figure 13. Simple boxplot of disease duration by segment


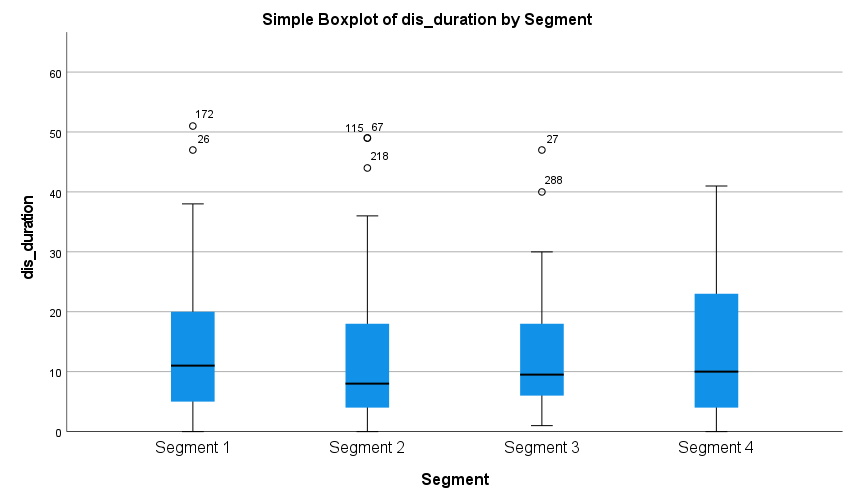


Figure 14. Bar chart Gender by segment


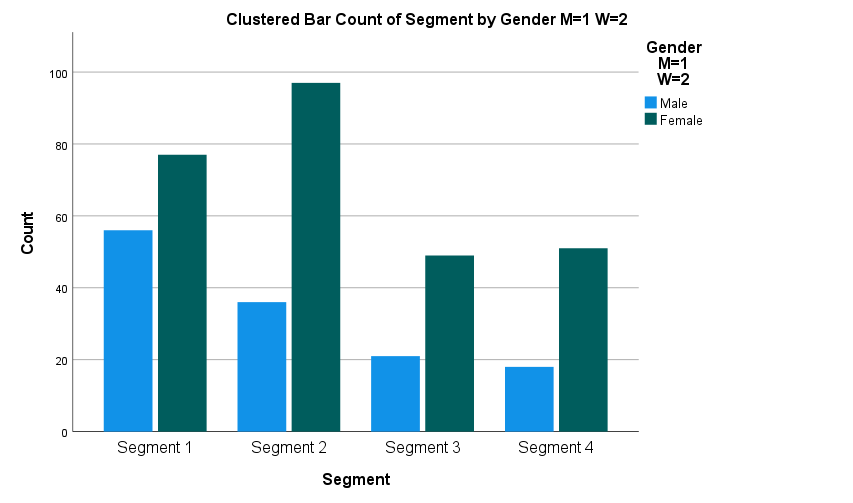


Figure 15. Bar chart MS type by segment


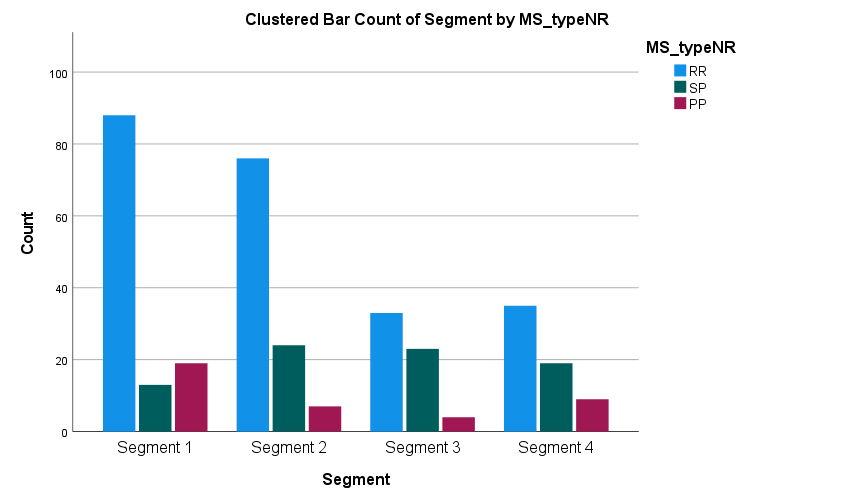

Supplement: Supplementary file 4 — Supporting Appendix: brb370634‐sup‐0004‐Appendix4.docx [file BRB3-15-e70634-s002.docx]
